# Exceptional discovery of a shallow-water hydrothermal site in the SW area of Basiluzzo islet (Aeolian archipelago, South Tyrrhenian Sea): An environment to preserve

**DOI:** 10.1371/journal.pone.0190710

**Published:** 2018-01-04

**Authors:** Valentina Esposito, Franco Andaloro, Simonepietro Canese, Giovanni Bortoluzzi, Marzia Bo, Marcella Di Bella, Francesco Italiano, Giuseppe Sabatino, Pietro Battaglia, Pierpaolo Consoli, Patrizia Giordano, Federico Spagnoli, Violetta La Cono, Michail M. Yakimov, Gianfranco Scotti, Teresa Romeo

**Affiliations:** 1 Dipartimento per il monitoraggio e la tutela dell’ambiente e per la conservazione della biodiversità, Istituto Superiore per la Protezione e la Ricerca ambientale (ISPRA), Milazzo, Italy; 2 Sezione Oceanografia - OGS, Istituto Nazionale di Oceanografia e Geofisica Sperimentale, Trieste, Italy; 3 Dipartimento per il monitoraggio e la tutela dell’ambiente e per la conservazione della biodiversità, Istituto Superiore per la Protezione e la Ricerca ambientale (ISPRA), Roma, Italy; 4 Stazione Zoologia Anton Dohrn, Napoli, Italy; 5 Istituto di Scienze Marine, ISMAR-CNR, Bologna, Italy; 6 Dipartimento di Scienze della Terra, dell’Ambiente e della Vita, Università degli studi di Genova, Genova, Italy; 7 Dipartimento di Fisica e Scienze della Terra, Università di Messina, Messina, Italy; 8 Istituto Nazionale di Geofisica e Vulcanologia, INGV, Palermo, Italy; 9 Istituto di Scienze Marine, ISMAR-CNR, Ancona, Italy; 10 Istituto per lo studio dell’Ambiente Marino Costiero, CNR, Messina, Italy; 11 Immanuel Kant Baltic Federal University, BFU, Kaliningrad, Russia; Northwest Fisheries Science Center, UNITED STATES

## Abstract

The geological, biological and geochemical features of a particular field of hydrothermal vents, discovered in the Panarea Volcanic Complex during a research survey carried out in 2015, are described for the first time. The site, located at 70–80 m depth off the South-western coast of the islet of Basiluzzo, was named Smoking Land for the presence of a large number of wide and high active chimneys and was characterized in terms of dissolved benthic fluxes, associated macrofauna and megafauna communities and preliminary mineralogy and geochemistry of chimney structures. On the whole field, a total of 39 chimneys, different in size and shape, were closely observed and described; 14 of them showed emission of low temperature hydrothermal fluids of marine origin characterized by acidified chemical conditions. The CTD and benthic chamber measurements highlighted that the Smoking Land is able to form a sea water bottom layer characterized by variable acidity and high DIC and trace elements concentrations; these characteristics weaken moving away from the chimney mouths. The SEM-EDS analysis of the collected solid samples revealed a chimney structure principally composed by amorphous and low crystalline Fe-oxyhydroxides of hydrothermal origins. The ROV explorations revealed a wide coverage of red algae (*Peyssonnelia* spp.) colonized by the green algae *Flabiella petiolata* and by suspension feeders, mainly sponges, but also bryozoans, and tubicolous polychaetes. Although novent-exclusive species were identified, the benthic communities found in association to the chimneys included more *taxa* than those observed in the surrounding no-vent rocky areas. These first findings evidence a submarine dynamic habitat where geological, chemical and biological processes are intimately connected, making the Smoking Land an important site in terms of marine heritage that should be safeguarded and protected.

## Introduction

The submarine hydrothermal activity plays a fundamental role in the life of the ocean as it drives many processes developing at the seafloor. The hydrothermal vents are well known to be clues of either ongoing or extinct volcanic-derived activities and indicate the presence of thermal energy sources underneath the seafloor, allowing the circulation of seawater through fractures of hot magmatic rocks. This apparently simple process is responsible for the recycling of many chemical elements from rocks to seawater. The peculiar microbial activity, which develops in and around the vents is often, particularly in the deep oceanic hydrothermal systems, the only living form able to feed on and grow using chemical elements instead of organic matter. The hydrothermal vents thus represent a tight and indissoluble link between rocks and life, organic and inorganic environments, on Earth. Although the presence of high-temperature vents and massive chimneys was formerly indicated for the deep ocean [1,2], the existence of hydrothermal systems located at shallow depths was documented in several volcanic areas around the world [3]. In the Mediterranean Sea active hydrothermal vents are located off some Greek islands [4] and along the Aeolian arc [5–7].

**Fig 1:**
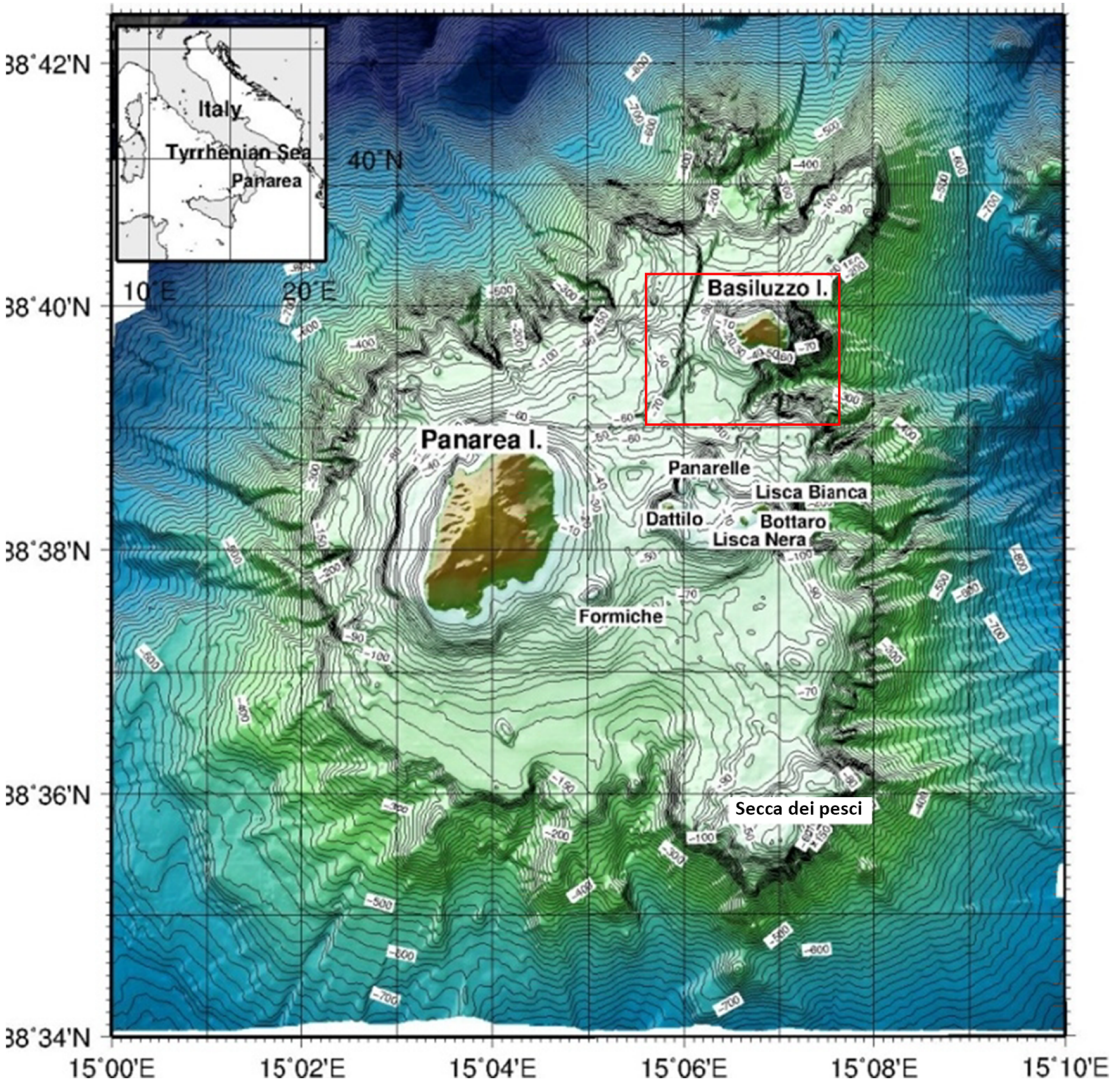
Study area. Map of the Panarea Volcanic Complex showing the investigated area (red rectangle). Bathymetries from ISMAR, reprinted from [24] under a CC BY license, with permission from ISMAR-CNR, original copyright 2013.

The whole submarine hydrothermal system of Panarea Island (Aeolian archipelago) is as wide (70 km^2^) as the Milos hydrothermal fields (Aegean Volcanic Arc) [8], is composed of a near-circular platform bounded by a shelf break at a depth of 120–130 m (Fig 1; [9]), and includes active vents marked by intense discharge of CO_2_-dominated gases and thermal fluids with temperatures as high as 140°C [10,11]. This hydrothermal system underwent an abrupt increase of the venting activity in November 2002 [12,13] due to the injection of magmatic fluids in the deep geothermal body, which caused a low-energy submarine explosion [14] killing almost all the living matter in the area (fish, corals, *Posidonia* etc). It was demonstrated that upraising of new magma at the nearby volcanic island of Stromboli allowed hot magmatic volatiles to migrate toward Panarea via the normal active fault linking the two volcanic edifices [15].

Hydrothermal processes producing sediment-hosted sulphide deposits and Fe-rich crusts and chimneys were also reported around the Basiluzzo dome [16–19]. Recent studies carried out over the North-Eastern area of the islet revealed the presence of a benthic community strongly dominated by the tube-dweller amphipod *Ampelisca ledoyeri* (Bellan-Santini & Kaim-Malka, 1977) that monopolizes the thin layer of fine sediment covering the gas-hydrothermal “low temperature” Fe oxyhydroxide precipitates from 80 to 120 m depth [20,21]. Moreover, in the steep slope at deeper bathymetries (> 180 m), Bortoluzzi et al [22,23] underlined the presence of rare sessile species associated with actively diffusing (diffusive ferruginous seep) and inactive vertical pinnacles and supported by complex and stratified microbial communities with a high proportion of ammonium- and iron-oxidizing chemoautotrophs.

Despite the numerous and detailed information existing on the geomorphologic features of the Panarea Volcanic Complex, the investigations on this submarine hydrothermal system are still ongoing with the aim of better constraining the hydrothermal system. A recent cruise carried out in 2015 revealed the presence of new structures with shapes different to that of the already investigated vents, made of chimneys of various size with diffused venting of gases and thermal fluids.

The present paper provides the first information about these peculiar hydrothermal structures discovered at 70–80 m depth off the South-western coast of Basiluzzo. The site was named “Smoking Land” for the presence of a large number of wide and high active chimneys.

The main targets are to investigate the geochemical features of the discovered chimneys and the associated biological assemblages, characterize the surrounding environment and provide some management indication to assure their protection. To reach these objectives, a multidisciplinary approach was used to describe this hydrothermal ecosystem, in terms of macro and megafauna communities, dissolved benthic fluxes as well as preliminary mineralogy and geochemistry of the outer portion of the chimney structures and their morphologies. In addition, integrated seafloor investigation techniques including multibeam bathymetry, high resolution side scan sonar, Remotely Operated Vehicle (ROV) investigations and physico-chemical water column property measurements have also been used.

### Geo-volcanological and tectonic settings

The Panarea Volcanic Complex represents the emergent part of a submarine composite volcano, belonging to the Aeolian Arc in the southern Tyrrhenian Sea, largely dismantled by erosion and neo-tectonic regime (Fig 1; [9]). The eruptive history of this wide volcanic system is divided into six successive eruptive epochs with periods of volcanic activity separated by quiescence stages, the last of which contributed to the emplacement of the Basiluzzo endogenous dome (54 ± 8 ka) [25,26]. Panarea and Basiluzzo islets are surrounded by an abrasion platform, similar in size to that of the other Aeolian Islands. To the east of Panarea, the islets of Basiluzzo, Dattilo, Panarelli, Lisca Bianca, Bottaro, Lisca Nera and Formiche form a small archipelago that emerges from the eroded submarine shelf at the top of the volcano [27–29]. Remnants of the primary volcanic structures, such as eruptive centers and lava domes, are traceable e.g. at Secca dei Pesci, to the S and SW area of Panarea and to the NW and NE of Basiluzzo [29]. The most remarkable active tectonic structure of the area is a NNE-SSW, NE-SW trending graben, located North-East of Panarea. The western boundary of the graben, consisting of a NNE-SSW, NE-SW trending extensional fault array, coincides with a series of evident escarpments, along which fresh volcanic rocks outcrop. Gas venting is frequent at the base and at the top of these faults. In the sedimented areas close to the faults, hydrothermalism is witnessed by the presence of white patches of sulfide mineralization and accumulations of Fe-rich ochre and red-colored sediments containing fragments of consolidated Fe-oxide crusts [29].

In the investigated area, the presence of hydrothermal activity had already been revealed by several authors [16–19]. Marani et al [16] and Gamberi et al [17,29] highlighted the presence in the area of gas venting, white patches, bacterial mats and Fe-oxyhydroxides precipitates relative to recent hydrothermal centered activity. Moreover, massive Ba-Pb-Zn enriched sulfide depositions were reported to the south of the islet of Basiluzzo along and near the eastern fault.

## Materials and methods

### Ethics statement

All the data collected in the present study have been gathered by using non destructive and non invasive sampling methods. No organism was collected, injured or manipulated. The research, from a formal point of view, has been funded and committed by the Sicilian Region with the aim of assessing the marine biodiversity of the hydrothermal systems in Sicilian waters.

Before sampling, specific authorizations were granted by the navy officer Mr. Giuseppe Salemme of the Sicilian Navy Headquarter (Marisicilia), by Mr Corrado Ascione of the MARISTAT Department, by the Italian Hydrographic Institute (MARIDROGRAFICO), by the Coast guard of Milazzo and the Lipari Maritime District Office. This study did not involve endangered or protected species.

### Methodological approach

The investigations were carried out combining on-board and laboratory analyses. The seafloor was carefully mapped by acoustic devices, and visual observations made by ROV, which also allowed the collection of solid samples and to perform *in situ* measurement of physico-chemical parameters. Water column properties were investigated by CTD and pCO_2_ measurements, dissolved benthic flux measurements have been carried out by benthic chamber deployments.

### Seafloor surveys and samples collection

#### Multibeam

The volcanic complex was mapped using a Kongsberg EM2040 multibeam sonar system operating at a frequency of 400 kHz and 300 KHz. Data were acquired with 40% lateral overlap using the Seafloor Information System (SIS) software and processed by means of HIPS and SIPS (Caris) software. 3D maps were created using Fledermaus (QPS) software.

#### ROV survey

**Fig 2:**
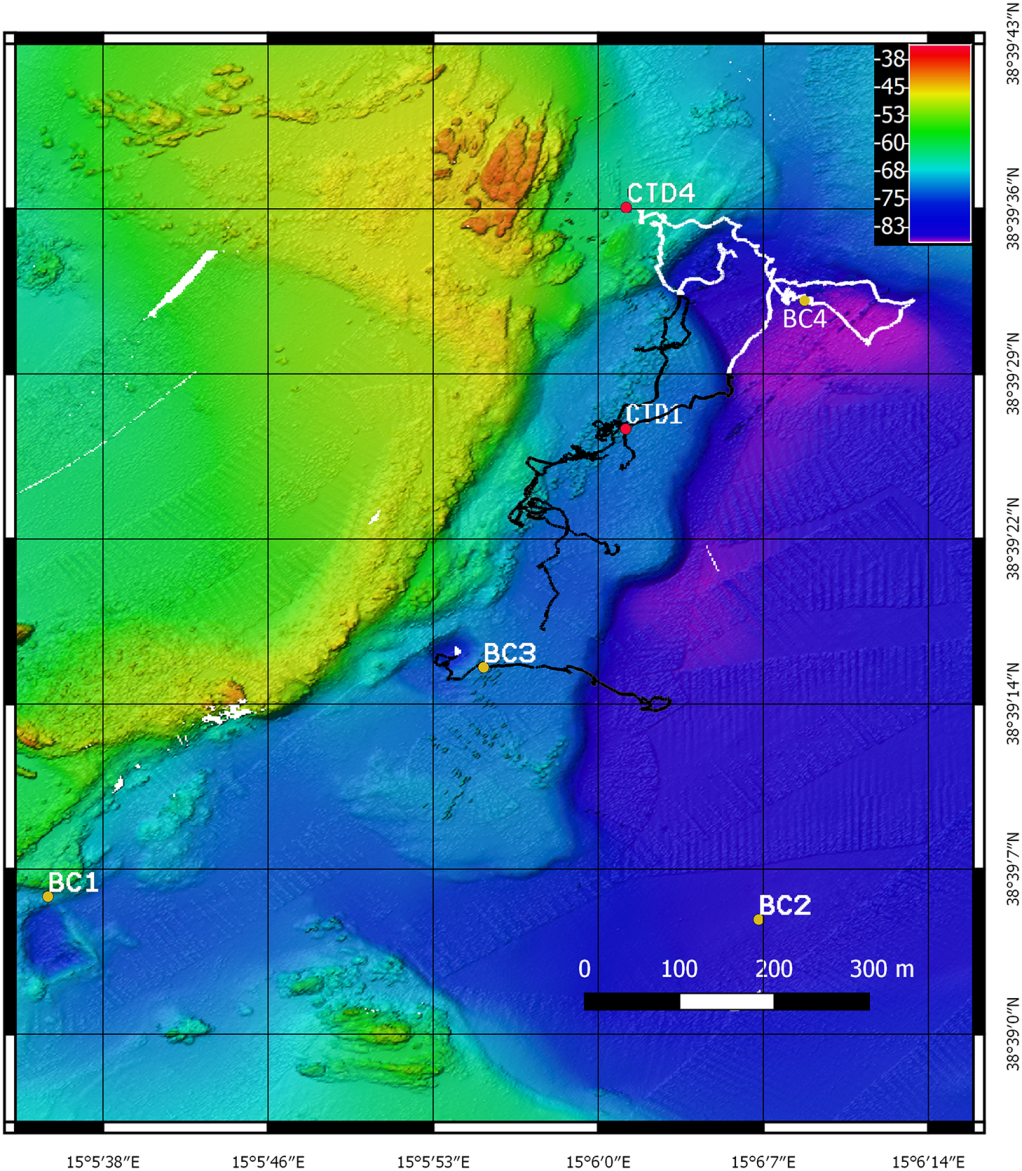
ROV tracks. Map of the investigated area reporting the tracks covered during the ROV survey. The black tracks covered the Smoking land; the white tracks covered the no-vent surrounding area.

**Fig 3:**
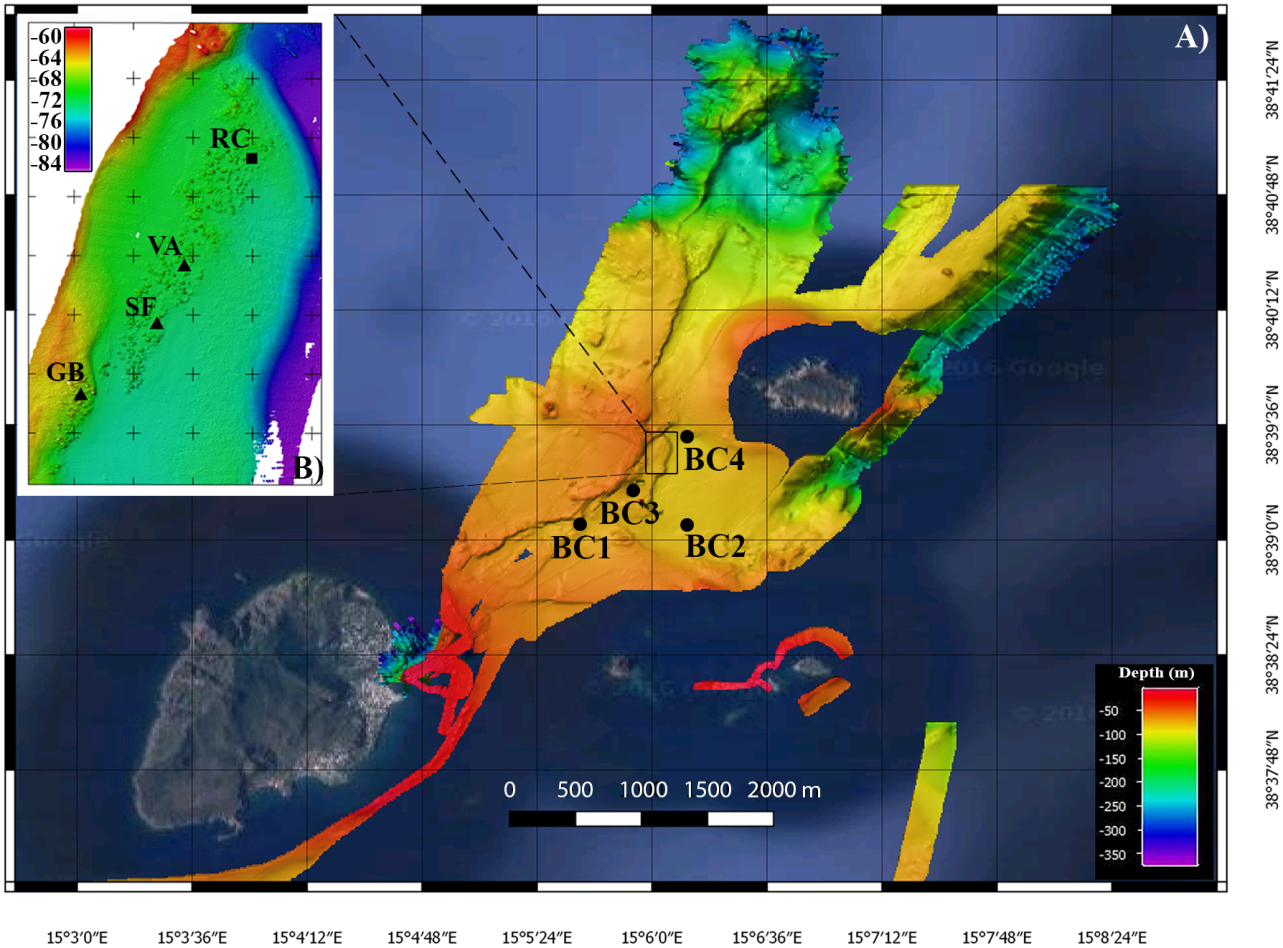
High-resolution swath bathymetry maps. A) map of the whole investigated area, SW of Basiluzzo Islet. The black dots indicate the benthic chamber stations; B) map of the Smoking Land hydrothermal field (WGS84, UTM33, cell size 20x20 cm, vertical exaggeration 3X). The black triangles indicate the location of the sampled chimneys, while the black square indicates the location of the chimney reconstructed based on HD video-images.

A fine scale ROV survey, covering the area mapped by the multibeam (Fig 2), was carried out to classify and characterize the hydrothermal structures and the associated fauna. The Pollux III ROV was equipped with a high resolution camera (Canon EOS 5D, 20 megapixel), two strobes (Canon), a high definition video camera (Sony HDR-HC7), 3 jaw grabbers, two parallel laser pointers to measure *in situ* seafloor structures and a USBL (Ultra Short Baseline—TrackLink 1500 MA Link Quest Inc.) underwater acoustic position system to accurately record the ROV’s geographic position. The main hydrothermal structures were identified and mapped. A small basket net was installed on the ROV to collect samples of terminal parts of chimneys. Samples of a total of three chimneys were collected, named VA (Chimney_VAlentina, 38°39’27”N, 15°6’1”E), SF (Chimney_Sagrada Familia, 38°39’25”N, 15°6’0”E), and GB (Chimney_Giovanni Bortoluzzi, 38°39’23”N, 15°5’57”E) (Fig 3B). In addition, a fourth chimney (RC, Chimney_ReConstructed, 38°39’30”N, 15°6’3”E) (Fig 3B) was reconstructed by means of a mosaic of 250 HD-video images processed with the Software Agisoft PhotoScan (www.agisoft.com) in order to provide a graphical representation of the investigated chimneys allowing a better understanding of the size and shape of the entire structure of the chimney.

#### pCO_2_ and CTD probes

A C-sense pCO_2_ sensor (600 m depth, measurement up to 2,000 ppm, Turner Designs) was used to measure the pCO_2_ concentrations in the habitats explored by ROV. In particular, pCO_2_ values were recorded at various depths and locations inside the Smoking Land habitat and in the fluids flowing outside the chimneys.

Moreover, an EXO2 Sonde CTD was used to characterize the water column environment of the Smoking Land and no-vent areas (Fig 2) by measuring the physico-chemical parameters (temperature, conductivity, dissolved oxygen, pH, ORP, turbidity, TDS (calculated) and salinity (calculated)), and to measure onboard the physico-chemical parameters of water inside the hydrothermal vents collected.

#### Benthic flux measurements

A Benthic Chamber (BC) was used to measure dissolved benthic fluxes [30]. The BC is a cylinder of plexiglass with the lower face open over the sea bottom and the upper face closed. The BC is able to confine a defined volume of water (approximately 100 liters) over a known area overlying the sediment. The BC is equipped with an internal stirring system to reproduce the intensity of the hydrodynamics near the seabed responsible for the formation of the diffusive benthic boundary layer. A multiparameter probe is placed inside the BC to continuously monitor temperature, pH, conductivity, dissolved oxygen, redox potential and salinity. The BC is equipped with a sampling system consisting of 8 pairs of syringes able to collect or inject a volume of 140 ml of water or tracer inside or outside the BC. The timing of sampling or injection is programmable depending on the operational needs. The BC was deployed on flat sea floor in four sites of the investigated area (BC1, BC2, BC3, BC4) (Figs 2 and 3A): BC1 (38°39’16"N, 15°5’55"E E) and BC3 (38°39’6"N, 15°5’36") were deployed near the N and NNE trending linear faults, next to the southern limit of the Smoking Land. BC2 (38°39’5"N, 15°6’7"E) was deployed on the SW zone of the depression and BC4 (38°39’32"N, 15°6’9"E) in the northern area of the depression. To calculate the dissolved benthic fluxes, the physico-chemical parameter (pH and oxygen) values were measured by the multiparametric probe inside the chamber, every five minutes, during each deployment, while Dissolved Inorganic Carbon (DIC) and trace element (Fe, Mn, Zn and Al) concentrations were determined inside the chamber at different times of each deployment (see S1 Dataset). Each benthic chamber deployment lasted between six and eight hours (see S1 Dataset). Water samples were collected every 1–1.33 hour. The benthic flux of oxygen, H^+^, DIC and trace elements were calculated by multiply the molar concentration for the eight of the benthic chamber, calculated from the dilution of a solution of CsCl injected in the camber (around 27 cm), and then dividing the result for the timing of the sampling [30].

For the H^+^, other than the flux, also the variations of pH units for surface and time unit have been shown (see Table 3 in the Results section) to give an idea of the decreasing of the pH with time during the benthic chamber deployment. For the uncertainties of the fluxes, the R^2^ (see Table 3 in the Results section) of the regression between the areal concentrations and the times of the sample collection or physico-chemical parameter measurements (see S1 Dataset) was considered. DIC concentrations were measured by coulometric method [31] with an error of 0.5% and a precision of ±σ = 0.9. Element concentration was measured by ICP-OES (Thermo iCAP6000) with an error of 5%.

### Analytical methods

#### Video, images and data analysis

Overall 8 dives providing over 7 hours of HD ROV-footage of the sea-bottom of the entire explored area (Fig 1; red rectangle) were analyzed to describe habitats and hydrothermal structures of the Smoking Land and the surrounding areas and to identify the associated communities to the lowest possible *taxon*.

A preliminary examination of the obtained videos showed that sampling rate of segments of 15 min allowed a good distinction between living communities associated to the two different type of vents identified (chimneys with and without emission). From 8 dives a total of 23 video segments were extracted as sample units: 19 video segments in the Smoking Land plus 4 in the no-vent area. Unusable videos (eg. out of focus, too far off the bottom) and videos of soft bottom in the no-vent area were removed from the dataset.

To allow a better identification of the *taxa* observed in the videos, a total of 354 HD photos were opportunely collected during the survey and analyzed.

Then, considering that the laser pointers were not always visible and that some of the major taxonomic groups were difficult to identify at specific level only by image analyses, the composition of living assemblages associated with the Smoking Land and with the surrounding no-vent area was compared through a non-parametric multivariate statistics performed on species presence/absence data recorded for each sample unit (see S2 Dataset).

Particularly, the Analysis of Similarities (ANOSIM) test was carried out on the basis of a Sorensen similarity index with 999 permutation. Then, a multivariate multiple permutation test (SIMPER) was also performed to determine the contribution of each species to the average dissimilarity between groups, in terms of Bray-Curtis similarities. Finally, the pattern of correlation among *taxa* and the different areas was investigated by the correspondence analysis. All these analyses were performed using the statistical software applications PRIMER6 & PERMANOVA+ [32,33] and the software package STATISTICA, version 10 [34].

#### Scanning electron microscopy—Energy dispersive spectrometry (SEM-EDS)

Measurements on the solid samples collected from the chimney walls and the bottom crusts were performed using an environmental scanning electron microscope, ESEMFEI Inspect-S, coupled with a spectrometer, Oxford INCA PentaFETx3 EDS, an Si (Li) detector equipped with an ultra-thin window ATW2, with a resolution of 137 eV at 5.9 keV. The spectral data were acquired under ESEM conditions at working distance of 10 mm with an acceleration voltage of 20 kV, counting times of 60 s, count per second 180 approximately 3000 cps with dead time below 30%. The results were processed by INCA software Energy. This software uses the XPP matrix correction scheme developed by Pouchou and Pichoir [35,36]. Moreover, an ESEM Zeiss EVO LS 10 with a thermo-ionic source (LaB_6_) coupled with the EDS Quantax (Bruker) with SDD was also used, the analyses were carried out on the bulk sample without any handling.

#### X-Ray powder diffraction

The X-ray powder diffraction analyses (XRPD) were performed using a Bruker D8 ADVANCE diffractometer with Cu K-alpha radiation on a Bragg-Brentano theta-theta goniometer, equipped with a SiLi solid-state detector, Sol-X. Acquisition conditions are 40 kV and 40 mA. Scans were obtained typically from 2° to 80° 2θ, with step size of 0.02° 2θ, with a count time of 1 second. Raw diffraction scans were stripped of ka2 component, 190 background corrected with a digital filter (or Fourier filter). Observed peak positions were matched against the ICDD JCPDS database.

#### X-Ray fluorescence spectrometry

The elemental composition was performed by X-ray fluorescence (XRF) spectrometry to determine the bulk-sediment chemistry of the rocks in terms of major, minor and trace elements by the WDXRF method with Bruker model S8 Tiger setup. The excitation source was a tube of Rh at 4 kW. To avoid detector saturation, power and current intensity were changed according to the analyzed element and its quantity. The concentrations of the major and minor elements have been calculated through the use of the software package GEO-QUANT M. For the calculation of the trace elements, however, the software GEO-QUANT T (a simple solution for the determination of these elements in geological materials) was used. The latter is a pre-calibrated and standardized method by the manufacturer, installed in the instrument present in the laboratory. This method was validated using two standard samples GBW07103 and GBW07406.

## Results

### Geomorphological features

The acquired swath bathymetry revealed an area characterized by the presence of a depressed region slightly elongated in a WNW-ESE direction with a depth of 80 m and an area of approximately 1.2 km^2^. To the west of the depression, two N- and NNE trending linear faults show outcrops of fresh rock (Figs 2 and 3A).

**Fig 4:**
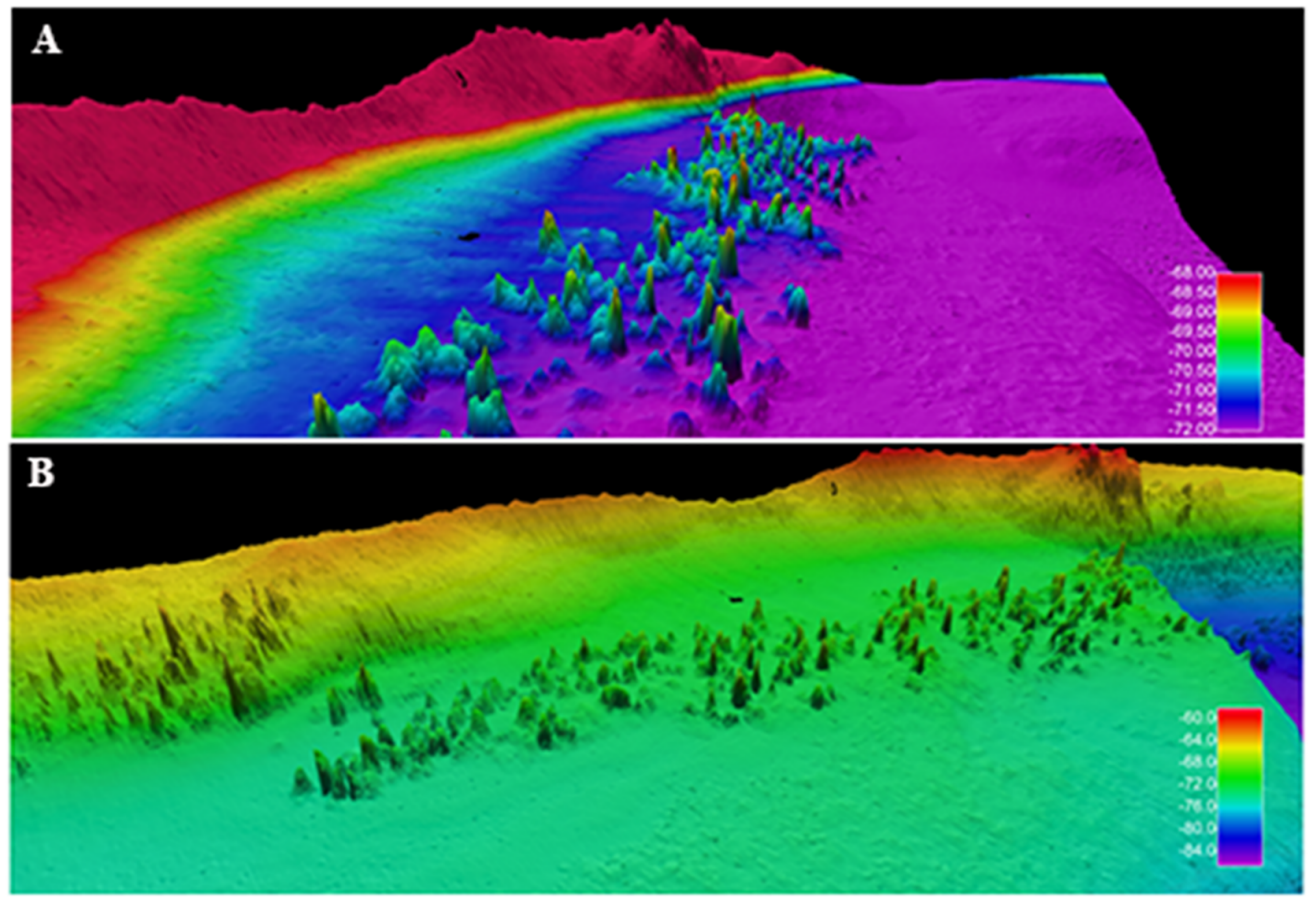
Three-dimensional model of the Smoking Land (WGS84, UTM 33, cell size 0.5 m). A) vertical exaggeration 2x; B) vertical exaggeration 3x.

**Fig 5:**
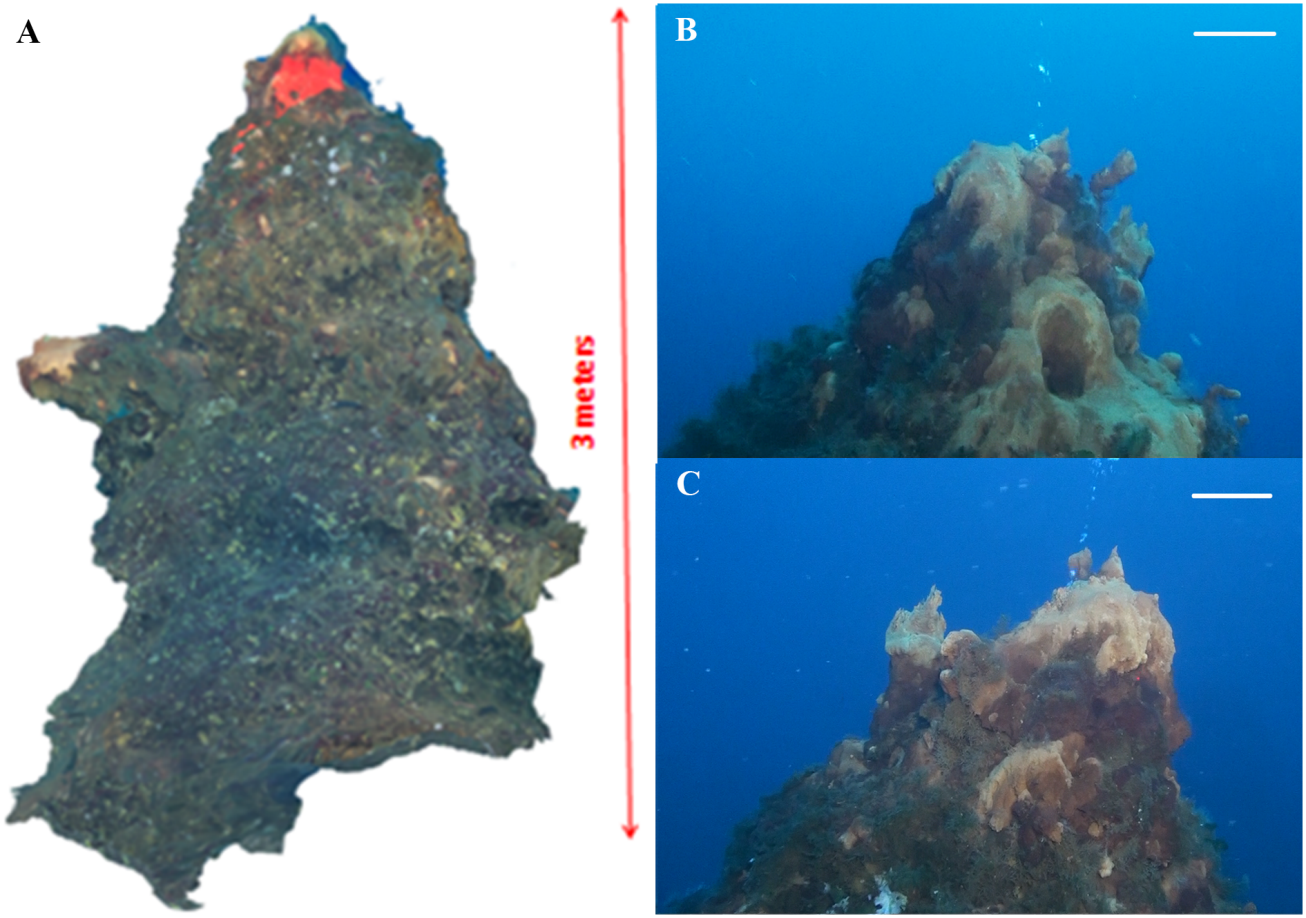
Images of a reconstructed chimneys located in the Smoking Land (38°39’30”N, 15°6’3”E; Fig 3: RC). A) Reconstruction of the chimney performed on HD video-image. B) underwater image of anterior side; C) underwater image of the posterior site of the summit of the chimney showing a wide yellowish deposit and the emission of bubbles. Scale bars: 20 cm for foreground.

In the area located at a depth of 70–80 m between the faults and the western limit of the depression, the new data acquired during the 2015 research survey revealed the presence of a never described vent field (0.5 km^2^), elongated in a NE-SW direction along the margin of the depression: the Smoking Land (Fig 3B). This site is composed by more than 200 hydrothermal chimneys, of various size (Fig 4) with a generally conic shape, inclination of 45–90°, height from 1 to 4 m and an average base diameter of about 3.8 m (n° = 68; standard deviation = 1.3 m) (Fig 5A).

### Characteristics of the chimneys

A total of 39 chimneys, different in size and shape, were described by means of ROV. On the whole, these complex structures were characterized by a wide biotic colonization and the presence of yellow to red precipitates, probably composed of iron oxyhydroxides (see below), distributed along the body of the chimneys or close to the upper and lateral mouths. Among all the observed chimneys, 14 showed clear effusive activity as revealed by the emission of bubble plumes or hydrothermal fluids, having visibly different density in comparison to the surrounding marine water (Figs 5B and 5C).

**Fig 6:**
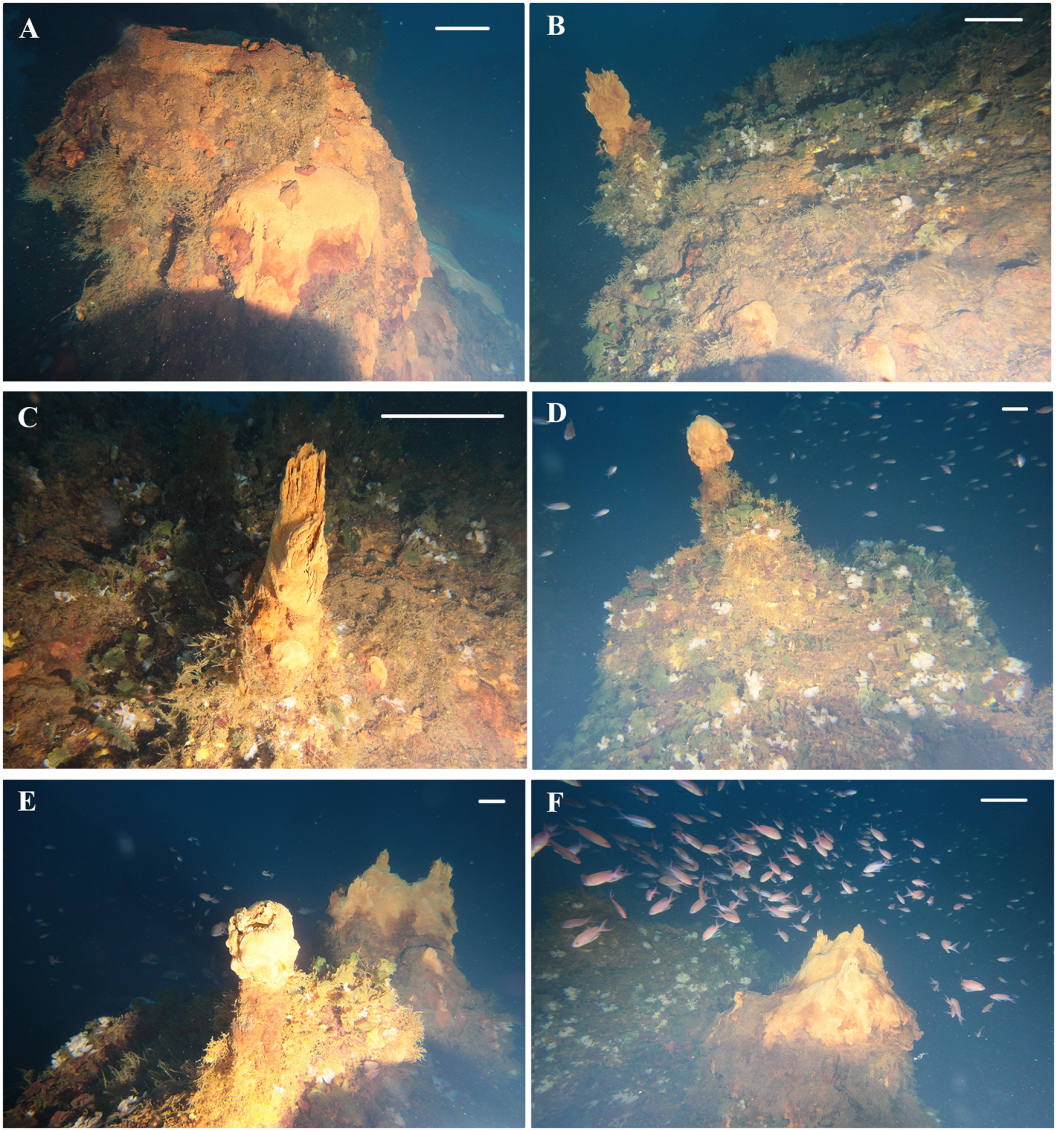
Underwater images of the chimneys located in the Smoking Land sampled during the 2015 research survey. (A) chimney VA; (B-C) chimney SF; (D-F) chimney GB. Scale bars: 20 cm for foreground.

The three sampled chimneys had the following characteristics:

VA: a blunted-cone shaped chimney, about 1 m high, located in the middle sector of the vent field (Fig 3B). It is characterized by a wide yellowish deposit corresponding to a lateral effusive mouth showing an intermittent release of bubbles. Smaller reddish deposits were observed all along the chimney body (Fig 6A).

SF: a short chimney, located at about 50 m south of VA (Fig 3B), that looks like a hill with a small comb-like yellowish deposit corresponding to a lateral mouth, releasing hydrothermal fluids with different density in comparison to the surrounding marine water (Fig 6B and 6C).

GB: a short but wide chimney, located at about 85 m south of SF (Fig 3B), showing two different deposits on the summit (Fig 6D–6F). The first one corresponded to an effusive mouth showing intermittent release of gas bubbles and hydrothermal fluids (Fig 6E). The second one, located on the other side of the vent summit, is characterized by three comb-like mouths, releasing hydrothermal fluids with different density in comparison to the surrounding marine water (Fig 6F). Smaller reddish deposits are observed all along the chimney body.

### Biological communities

The analysis of the ROV video and images led to the identification of a total of 44 benthic *taxa*, including algae, invertebrates and fish, 31 of which were found to be associated to the observed vents (Table 1).

**Table 1:** List of identified *taxa* and number of video segments in which each *taxon* was observed associated to chimneys of Smoking Land with and without evidences of fluid emissions and to the surrounding rocky no-vent area.

Taxa	code	chimneys WITH fluid emission	chimneys WITHOUT fluid emission	NO-VENT area
**total number of video segments analyzed**		**8**	**11**	**4**
**Chlorophyta**				
*Codium bursa*	Cod_bu	−	1	−
*Flabellia petiolata*	Fla_pe	7	8	1
**Rhodophyta**				
*Peyssonnelia* sp	Pey_sp	6	10	−
Corallinales	Cora	−	−	4
**Porifera**				
*Axinella* sp	Axi_sp	−	1	−
*Oscarella* sp	Osc_sp	−	1	−
cfr *Haliclona* sp	Hal_sp	1	−	−
cfr Haplosclerida	Hapl	7	11	−
cfr Poecilosclerida	Poecil	1	1	−
Dictyoceratida	Dict	1	−	−
**Anthozoa**				
*Caryophyllia* sp	Car_sp	1	−	3
Clavulariidae	Cla	1	2	−
*Eunicella singularis*	Eun_sin	−	−	1
cfr *Paracyathus pulchellus*	Par_pul	−	−	1
**Hydrozoa**				
Campanulariidae (cfr *Clytia* sp)	Camp	1	−	−
Eudendriidae	Eud	2	−	−
**Echiurida**				
*Bonellia viridis*	Bon_vir	−	−	1
**Polychaeta**				
Sabellidae	Sab	4	5	2
Terebellidae	Ter	3	1	
*Protula* sp	Pro_sp	−	−	2
**Mollusca**				
*Bolma rugosa*	Bol_rug	1	−	−
Muricidae	Muri	−	1	−
**Bryozoa**				
*Hornera frondiculata*	Hor_fro	2	−	−
*Reteporella grimaldii*	Ret_gri	7	9	1
*Myriapora truncata*	Myr_tru	−	−	4
**Echinodermata**				
*Centrostephanus longispinus*	Cen_lon	1	1	4
*Hacelia attenuata*	Hac_at	1	−	1
*Holothuria forskali*	Hol_for	1	−	2
*Holothuria* sp	Hol_sp	−	−	1
*Stylocidaris affinis*	Sty_aff	−	−	2
*Marthasterias glacialis*	Mar_gla	−	−	1
*Chaetaster longipes*	Cha_lon	−	−	1
**Tunicata**				
*Clavelina dellavallei*	Cla_del	1	4	−
*Diazona violacea*	Dia_vio	1	−	−
*Halocynthia papillosa*	Hal_pap	−	−	1
*Ciona* sp	Cio_sp	−	−	1
**Vertebrata**				
*Anthias anthias*	Ant_an	3	3	2
*Lappanella fasciata*	Lap_fa	−	1	−
Gobiidae	Gob	−	2	2
*Gobius kolombatovici*	Gob_kol	−	4	1
*Gobius vittatus*	Gob_vit	−	1	−
*Serranus cabrilla*	Ser_cab	1	−	2
*Coris julis*	Cor_jul	−	−	1
**Others**				
unid. non-calcified organisms	uni_noncalc_org	7	10	−

**Fig 7:**
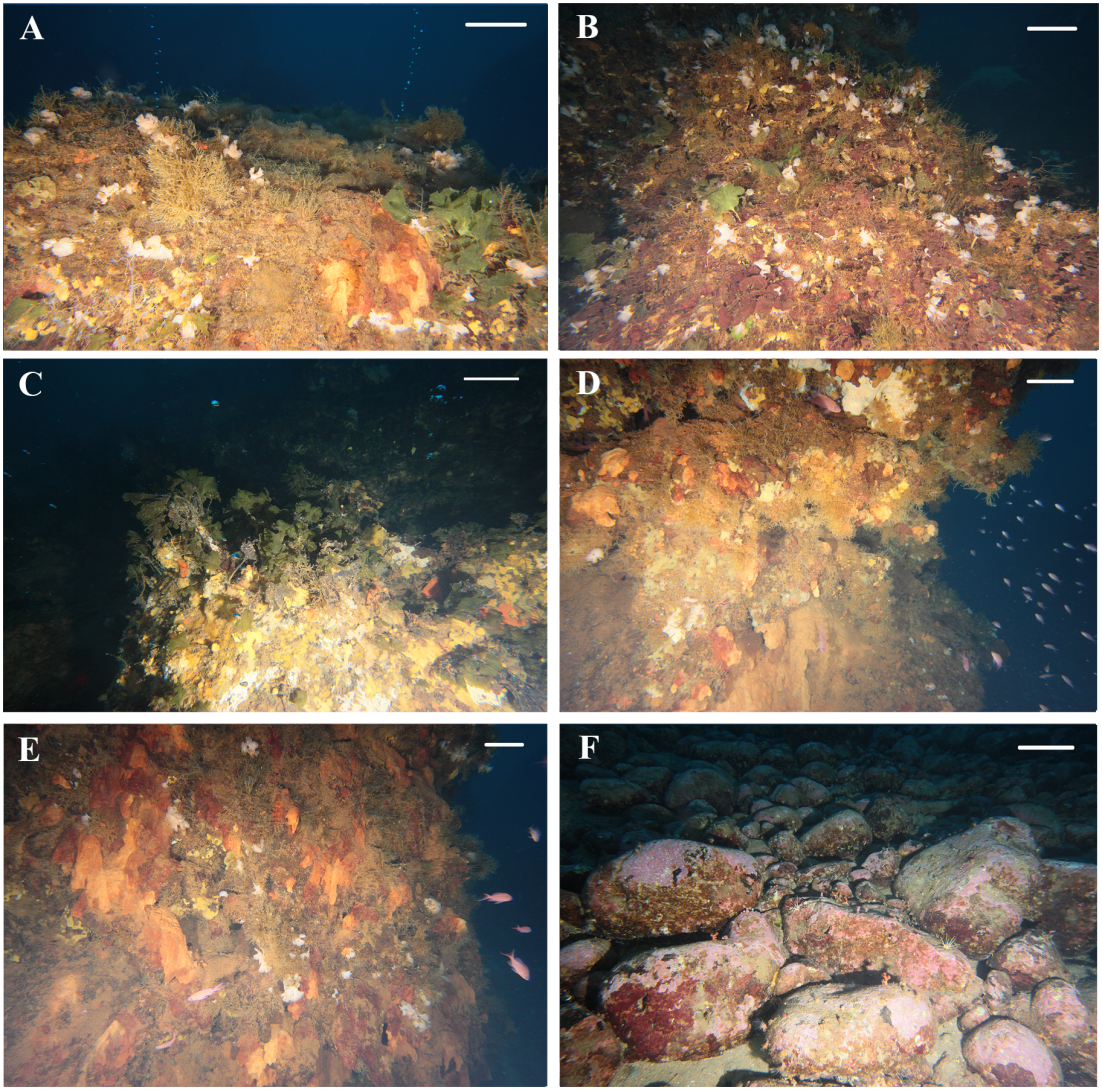
Underwater pictures of the Smoking Land vent fauna. (A) some colonies of *Reteporella grimaldii* and group of polychaetes Sabellidae on a chimney with active emissions of bubble plumes; (B) coverage of the red encrusting coralline algae *Peyssonnelia* spp. together with *R*. *grimaldii* and the green algae *Flabellia petiolata*; (C) emission mouth surrounded by *F*. *petiolata* and encrusting sponges (cfr. Haplosclerida and cfr. *Haliclona* sp.); (D-E) macrobenthic and fish community typically found to be associated with the investigated chimneys; (F) accumulation of volcanic rocks encrusted by red algae and colonized by some specimens of *Myriapora truncata*, *Stylocidaris affinis* and *Caryophyllia* sp. in the no-vent area North-West of the vents field. Scale bars: 20 cm for foreground.

Overall, the biological community associated with the Smoking Land is comparable to that of a coralligenous assemblage (Fig 7). In general, the chimneys showed an extended coverage of red algae (*Peyssonnelia* spp.) interrupted only by deposits and effusive mouths. Near the summit of the vents, this coverage appeared to usually be colonized by the green algae *Flabellia petiolata* (Turra) Nizamuddin, 1987, while, in the lower portions the benthic community resulted dominated by suspension feeders, principally sponges, but also bryozoans, and tubicolous polychaetes (Fig 7B, 7D and 7E). As reported in Table 1, of the identified 31 *taxa* observed in the Smoking Land, 23 were found in association with chimneys with active fluid emissions, while 20 were observed in association with chimneys without active emissions. Only 13 of the *taxa* were found in association with both types of chimney. Among the latter, the most commonly observed, all along the chimney bodies, were erect organisms such as the bryozoan *Reteporella grimaldii* (Jullien, 1903), observed in almost all the video segments on both chimneys with and without emission, mainly found in the lower part and sometimes close to the emission mouths (Fig 7A and 7B). Porifera of the Order Haplosclerida and polychaetes (Family Sabellidae) were found all over the chimney bodies, as well as non-calcified organisms, that need further investigation to be identified and were frequently found all around or on the top of the deposits and close to the emission mouths (Fig 7A and 7C). Overall 11 *taxa* were exclusively found in association with the chimneys with fluid emissions, while 7 were found only on the chimneys without emissions. However, all of these species were only occasionally observed.

The explored no-vent area, located close to the northern and southern margins of the Smoking land, is represented by the western zone of the depression revealed during the ROV survey. It is characterized by a relatively flat topography (80 m depth) and different seafloor conditions, including coarse and detritic sands with emerging rocks, and Fe-rich crust covered by fine sediments with no evidence of hydrothermal emissions.

To the North-West, the depression is delimited by a gently sloping edge, 70–80 m depth, consisting of lava, tuff deposits and stacked accumulations of volcanic rocks probably outcropped from the nearby trending linear faults (Fig 7F). This rocky bottom was chosen as control area for the characterization of the benthic community.

The living community associated to this habitat resulted composed by 24 *taxa*, among which the Echinodermata, represented by seven species, resulted as the most diverse group, with *Centrostephanus longispinus* (Philippi, 1845) being the most commonly observed species together with the bryozoan *Myriapora truncata* (Pallas, 1766) (Table 1).

Results of the ANOSIM test showed significant differences (p<0.01) between the living assemblages associated with chimneys of the Smoking Land and the surrounding no-vent area, while no differences were recorded inside the Smoking Land between chimneys with and without emissions. SIMPER tests showed the highest average dissimilarity between chimneys with emission and no-vent area communities (δ = 87.42), mainly ascribable to *M*. *truncata*, exclusively observed in no-vent areas, and Porifera of the order Haplosclerida, exclusively observed as being associated with chimneys.

**Fig 8:**
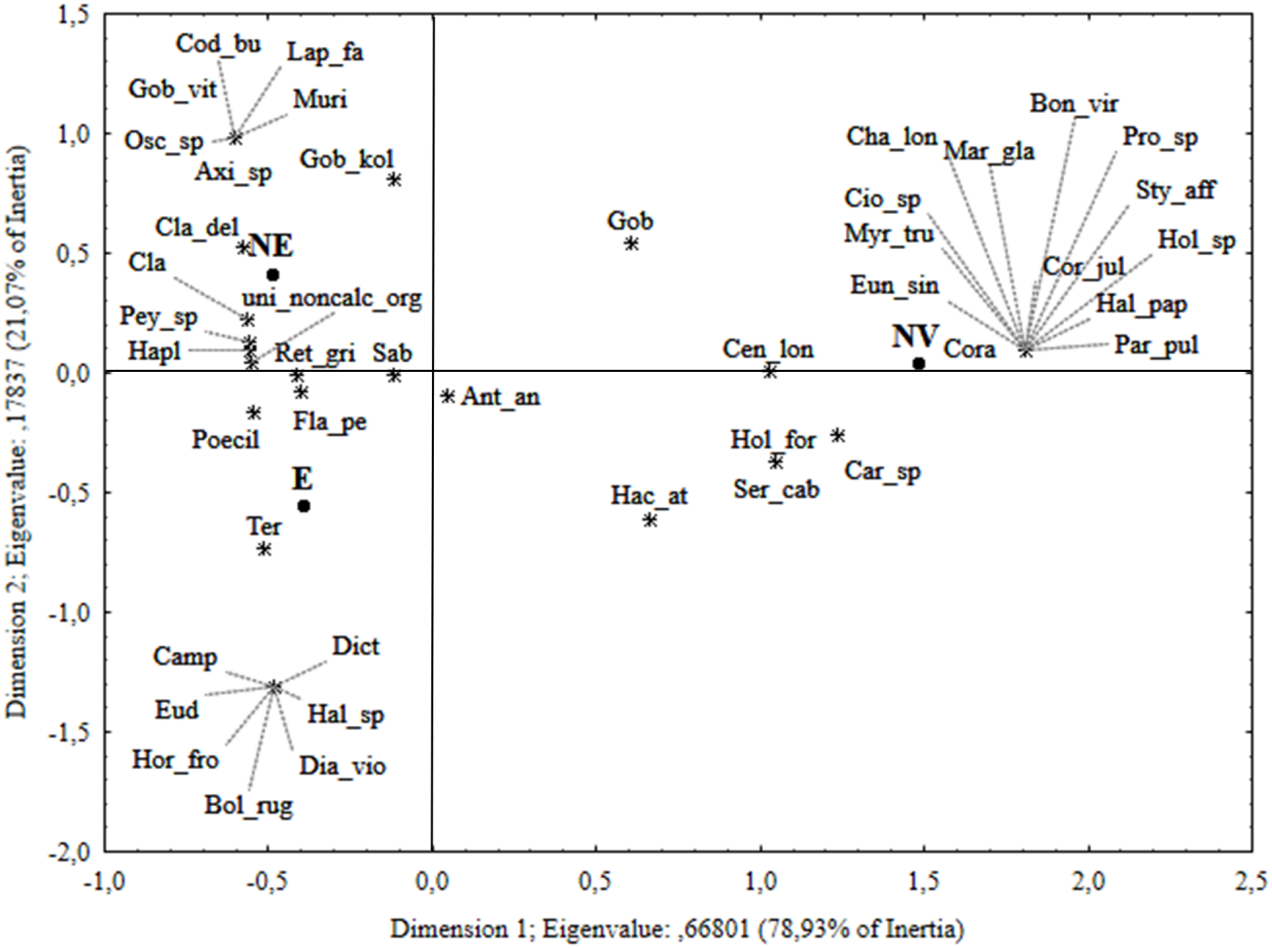
Correspondence analysis (CA) for area. Ordination diagram for the first two canonical axes of the correspondence analyses using species n. of observation data per area (E, chimneys with emission; NE, chimneys without emission; NV, no-vent area). Species codes as in Table 1.

For correspondence analysis (Fig 8), the first two dimensions explained 100% of the total variance. The first dimension showed a clear separation of no-vent areas (NV) from chimneys of Smoking Land, mainly related to the presence of calcified organisms such as echinoderms (*Marthasterias glacialis* (Linnaeus, 1758), *Chaetaster longipes* (Retzius, 1805)), anthozoans (*Eunicella singularis* (Esper, 1791), *Paracyathus pulchellus* (Philippi, 1842)) and serpulids (*Protula* sp.), exclusively found in NV. The second dimension showed a clear distinction between the living communities associated to chimneys without emissions (NE: upper left of the diagram) and chimneys with emissions (E: bottom left), ascribable to the species occasionally observed in association with only one of the two categories (Fig 8; Table 1).

### Physico-chemical parameters

**Fig 9:**
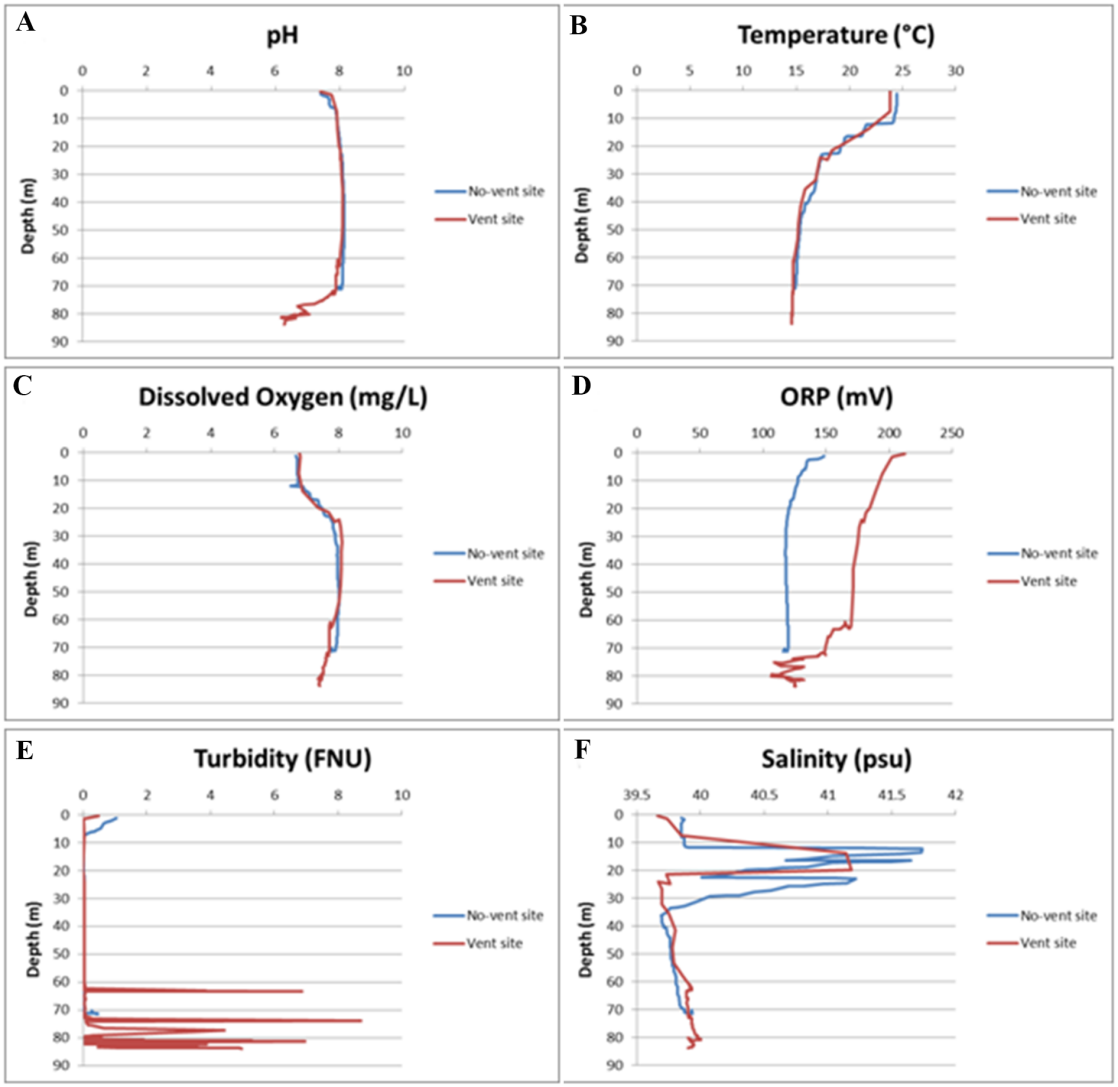
Physico-chemical parameters recorded by the EXO2 Sonde in the Smoking Land area. A) pH; B) Temperature; C) Dissolved Oxygen; D) Oxidation-Reduction Potential; E) Turbidity; F) Salinity.

The CTD investigations in the water column overlying the Smoking Land (CTD1; Fig 2) showed a three layer pattern (Fig 9, venting site) with a warmer and lesser oxygenated surface level and a bottom layer characterized by a general decrease of the pH and ORP and an increase of the turbidity.

In particular, in the Smoking land, the pH increased from 7.70–7.95 near the surface to 8.03–8.1 from 20 to 30 m depth, then it began to slightly decrease from 8.07–8.12, from 40 to 50 m depth, to 7.89–8.06 at 62–70 m depth and then it strongly decreased near the sea bottom, reaching the minimum acidic values of 6.19–6.96 at 69-81m depth (Fig 9A).

Temperature showed a warmer surface layer in the first 7.5–13 meters, with values ranging from 24.3 to 23.7°C, a mixed intermediate layer up to 35–38 m depth, where temperatures ranged from 15.5 to 15.8°C, and a bottom layer, with quite constant temperatures, reaching the minimum (14.5°C) at the bottom (Fig 9B).

Oxygen showed a lower concentration layer at the surface (6.68–6.79 mg/L up to 13.1 m depth), an higher concentration intermediate stratum (7.8–8.11 mg/L from 23 to 68 m depth) and a slightly lower bottom water level (7.4–7.7 mg/L near the bottom) (Fig 9C).

Also ORP showed a three-layer pattern with moderate increase in a surface layer and a noticeable decrease near the bottom (from 268–135 mV at 62 m depth to 98–114 mV) (Fig 9D).

Turbidity showed only some scattered increases both in depth and values over the 61 m depth (Fig 9E).

Salinity showed an intermediate increasing layer and slightly increasing values (from 39.67 to 39.99 psu) near the sea bottom (Fig 9F).

The CTD measurements carried out in the no-vent area (CTD4; Fig 2) recorded differences only in the pH, that showed constant values near the bottom (around 8.06, Fig 9A, no-venting site), and in the ORP that was lower over all the profile (Fig 9D).

The pCO_2_ sensor, mounted on the ROV, recorded very high pCO2 concentration values in the water column close to the chimney spills (from ~601 to ~1800 ppm) and off scale values (2,000 ppm) in the fluids escaping from the vents, while, in areas far from venting sites, pCO_2_ values ranged between 390 and 460 ppm.

The on-board measurements made by the EXO2 probe in fluid vent waters, collected together with the chimney samples by a ROV device, indicated the leach of a strong acidic fluid with no appreciable salinity variations in comparison to the surrounding sea-water (Table 2).

**Table 2:** Results of physicochemical parameter analyses carried out on board by the CTD probe in water samples collected in the hydrothermal water flux coming out of the GB and SF chimneys.

Parameter	Unit	GB	SF
Conductivity	μS/cm	52637.6	54909.8
TDS	mg/L	38530	38477.0
Salinity	psu	39.7	39.7
Dissolved Oxygen	% sat	71.1	99.6
	mg/L	5.19	7.0
pH	Units	4.9	5.6
ORP	mV	166.6	200.2
Turbidity	FNU	1726.9	169.2

With regard to the DIC concentrations, they varied from 3160.51 to 5279.06 mmol/L in the Smoking Land and are around 2300 mmol/L in the no-vent areas.

### Benthic fluxes measurements

Benthic flux measurements were carried out by the deployment of a benthic chamber in sites close to the Smoking Land and on the Graben Plain and borders (Figs 2 and 3A).

In and around the Smoking Land the measured benthic fluxes indicated a patch pattern with stations characterized by a strong release of acidic fluids enriched with DIC and trace elements, and other stations where the fluid releases were negligible (Table 3). In particular, a very high benthic flux from the bottom of DIC, protons and trace elements such as Fe, Mn, Zn and Al (Table 3), and a strong oxygen consumption have been measured in a site close to the Smoking Land area (station BC3, Table 3, Figs 2 and 3A). The high release, in localized areas of the seafloor, of acid fluids rich in DIC and trace elements is attributed to a sub-seafloor sea water circulation: the seawater, after penetrating into the sub-sea bottom, encounters and adsorbs rising volcanic CO_2_ produced by deeper hydrothermal activity of the Panarea Volcanic Complex, with following the reaction:

$${CO}_{2\;(aq)\;}+\;H_{2}O\;H_{2}CO_{3}\;{HCO}_{3}^{-}\;+\;H^{+}{CO}_{3}^{2-}\;+2\;H^{+}$$

**Table 3:** Dissolved benthic fluxes measured by the benthic chamber in the Smoking Land and in no-vent areas. pH has been shown both as flux of H^+^ and as variations of pH units per square meter per day during the measurements.

Station	Fluxes															
chemical	DIC	R^2^	Fe	R^2^	Mn	R^2^	Zn	R^2^	Al	R^2^	H+	R^2^	pH	R^2^	Oxygen	R^2^
units	mmol/m2*d		mmol/m2*d		mmol/m2*d		mmol/m2*d		mmol/m2*d		mmol/m2*d		unit/m^2^*d		unit/m^2^*d	
BC1	-19.29	0.024	−		−		−		−		-0.0024	0.551	0.3372	0.678	-0.77	0.036
BC2	-17.99	0.022	0.013	0.024	-0.006	0.997	−		−		-0.0115	0.588	0.4615	0.676	3.93	0.449
BC3	3223.90	0.951	16.477	0.978	0.735	0.991	0.288	0.889	1.536	0.985	1.7536	0.99	-1.0267	0.733	-52.92	0.98
BC4	-4.41	0.001	−		−		−		−		-0.0011	0.953	0.1706	0.968	-2.97	0.746

-: analysis not done because of negligible or not reliable flux.

In this way the sea-water becomes strongly acidic, dissolves the metals included in the sub-seafloor rocks, and then rises and flows out of the sea bottom along preferential paths. In the submerged Panarea Volcanic Complex the preferential paths and flowing out sites are localized along the two fault plains of the graben flanks to the North-East of Panarea Island.

Conversely, deployments of the benthic chamber in the no-vent sites located in the flat seafloor of the depression (stations BC1, BC2 and BC4 of Figs 2 and 3A) recorded negligible dissolved benthic fluxes of DIC, H^+^, Fe, Mn and oxygen (Table 3).

This patch pattern is attributed to the formation of Fe-oxyhydroxide hardgrounds at the surface of seafloor that formed an impermeable layer for the hydrothermal fluxes from the bottom. The Fe-oxyhydroxide hardground formation was deduced from the cores and box-cores recovered on flat graben seafloor during previous surveys, that showed an intense bubbling after the recovering. This strong bubbling was attributed to the breaking of the superficial Fe-oxyhydroxide layer, that appeared as an hard reddish ten centimeters thick surface layer over a complex sedimentary aggregate. The Fe-oxyhydroxide main composition of this surface layer was inferred by the SEM-EDS analyses of samples collected in it.

**Fig 10:**
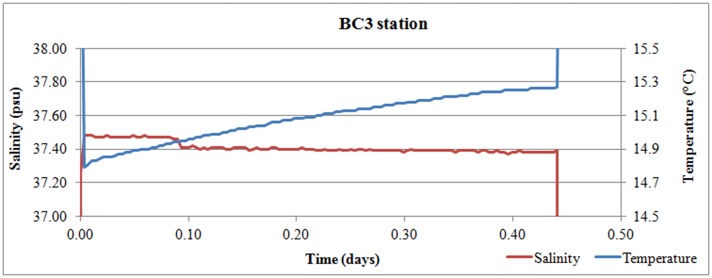
Benthic chamber measured parameters in BC3 site. Temperature and salinity recorded in the Benthic Chamber in the BC3 site located close to the Smoking Land.

The temperature and salinity recorded in the benthic chamber in the site of active leaking located close to the Smoking Land (BC3) and also in other venting sites on the graben plain did not show evidence of increase or decrease (Fig 10), excluding hot or hypersaline solution releases.

### Mineralogical and geochemical features of vents

Samples from the external wall of the VA, SF and GB chimneys, considered as representative of the Smoking Land hydrothermal field, showed an orange color with grey to black coatings, with very fine grain size and colloidal appearance.

**Fig 11:**
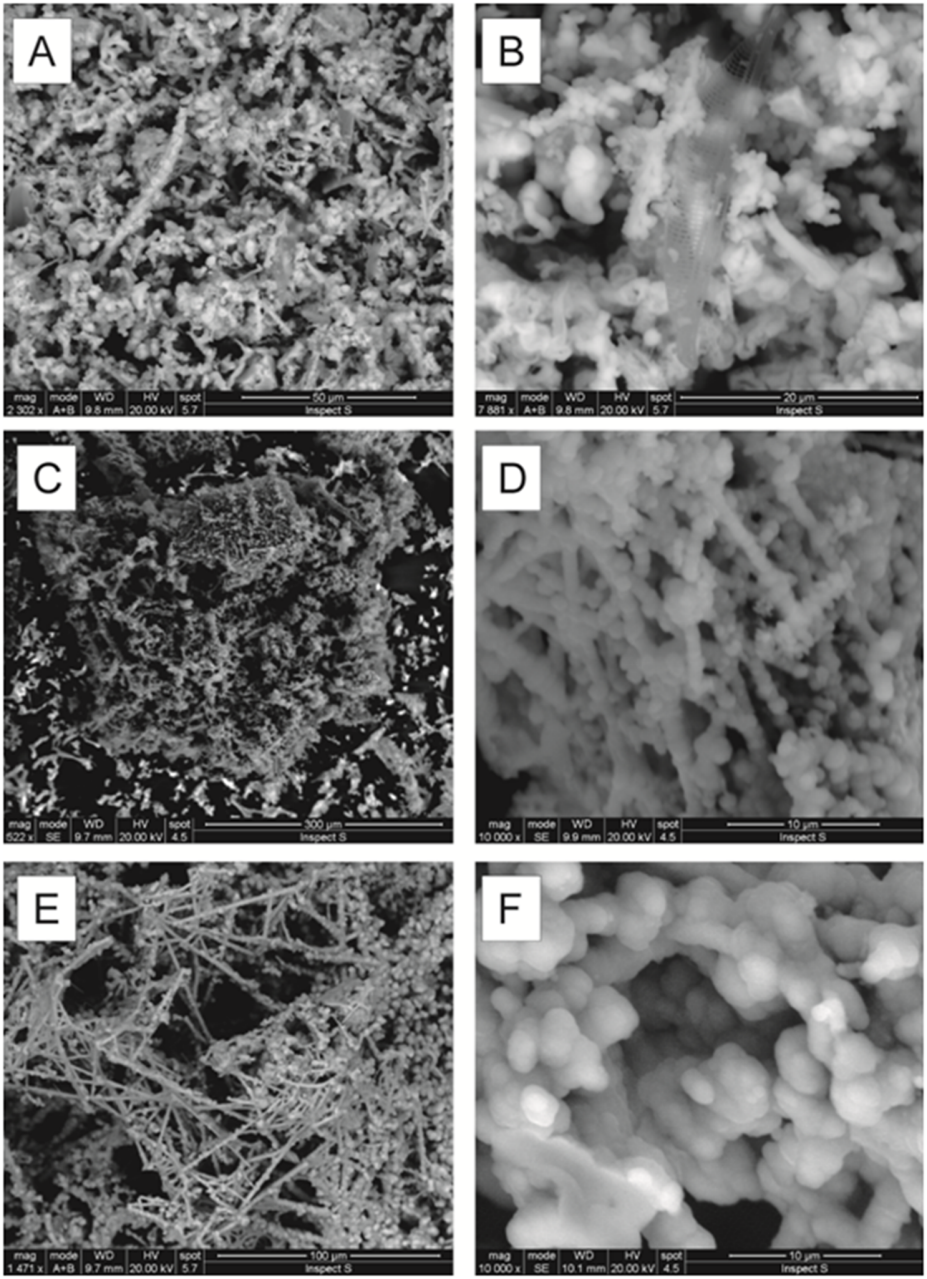
Scanning electron microscopy images of textures of the analyzed hydrothermal chimneys. (A-B) chimney GB; (C-D) chimney VA; (E-F) chimney SF.

Results of XRPD and SEM-EDS analyses demonstrated homogeneous features for all the investigated samples. The SEM-EDS analyses of textures revealed the presence of delicate structures such as non-helical filaments (rod) and spheres (cocci) and their aggregates. Filament morphologies were particularly variable and ranged from short simple-branching filaments (Fig 11A–11D) to complex filament networks (Fig 11E and 11F) sometimes remarkably similar to bacterial-like structures (Fig 11A–11F) in agreement with the results on the microbial activity of the Panarea submarine hydrothermal system [5,37]. SEM-EDS analyses (Table 4) revealed the presence of Fe-rich compositions (from 72.23–92.36 wt %) with slightly variable silica amounts (7.64–22.54 wt %) and low amounts of other oxides such as P_2_O_5_ (3.37–5.73 wt %) and CaO (0.67–0.96 wt %).

**Table 4:** SEM-EDS representative data (% wt) of the analyzed chimneys samples.

Samples	VA	VA	SF	SF	GB
**SiO**_**2**_	10.17	7.64	12.75	13.1	22.54
**P**_**2**_**O**_**5**_	-	-	5.73	3.65	4.47
**CaO**	0.89	-	0.96	0.89	0.77
**FeO**	88.94	92.36	80.63	82.36	72.23
**Total**	100	100	100	100	100

XRF data (Table 5) of major element confirmed the results of the SEM-EDS analyses and highlighted for all the analyzed vents a composition made of prevalent Fe_2_O_3_ (81.98–87.24 wt %) and SiO_2_ (6.78–9.23 wt %), with minor amounts of Na_2_O (2.19–2.78 wt %) and very low concentrations (≈ 1 wt %) of the other oxides (Al_2_O_3_, CaO, MgO, TiO_2_, P_2_O_5_, MnO, SO_3_). Trace elements composition was instead characterized by higher values of Mo (70; 225 ppm), Sr (85; 170 ppm), As (35; 108 ppm), V (51; 99 ppm), and Pb (31, 84 ppm), with minor amounts of Ni (26, 35 ppm), Y (24, 26 ppm) Rb (15, 22 ppm), Cu (20, 21 ppm), Zn (14, 18 ppm), Ba (11, 17), and U (6, 11 ppm); Sc, Nb, Ce and Th amounts resulted < 5 ppm in both the analyzed chimneys (Table 5).

**Table 5:** XRF data of major oxides (% wt) and trace elements (ppm) of the analyzed samples.

Major Oxides			Trace elements		
Sample	VA	SF	Sample	VA	SF
% wt			ppm		
**SiO**_**2**_	7.25	8.65	**Cd**	5	0
**Al**_**2**_**O**_**3**_	0.14	0.13	**Sc**	0	3
**Fe**_**2**_**O**_**3**_	87.24	84.12	**V**	69	51
**CaO**	0.48	0.56	**Ni**	26	35
**MgO**	0.39	0.43	**Cu**	21	20
**Na**_**2**_**O**	2.68	2.56	**Zn**	18	14
**K**_**2**_**O**	0.19	0.24	**As**	82	108
**TiO**_**2**_	0	0.001	**Rb**	15	22
**P**_**2**_**O5**	0.43	1.41	**Sr**	113	170
**MnO**	0	0	**Y**	26	24
**SO**_**3**_	0.06	0.02	**Nb**	1	2
**Cl**	1.04	1.02	**Mo**	210	70
**TOT**	99.90	99.14	**Ba**	11	17
			**Ce**	0	1
			**Pb**	31	84
			**Th**	3	2
			**U**	11	6

## Discussion

The present study provides the first description, in terms of structures and associated macro and megabenthic communities, of a new hydrothermal habitat in the Aeolian arc named the Smoking Land. The discovery of this vent field supplies new and important information on the minero-geochemical and biological features of the shallow-water hydrothermal systems of the Mediterranean Sea.

The origin of the Smoking Land, with all its numerous and large chimneys, can be attributed to the presence of a strong hydrothermal circulation along the North-western graben fault plane. Acidic fluids of marine origin, enriched in Dissolved Inorganic Carbon (DIC) and trace elements by volcanic degassing, leak from the bottom around the surrounding flanks of the depression and from the vents, accompanied in some cases by CO_2_ bubbling and in some others by apparent inactivity. As showed by the CTD and benthic flux measurements, the emitted fluids are not more saline or hotter than the surrounding marine water, in contrast to the hot subaerial Panarea emissions [38]. This suggests a rising of CO_2_ from deep volcanic rocks that triggers a sub-bottom sea water circulation and rock metal dissolution caused by the acidic solution conditions.

The variability in the fluid emissions observed in the chimney mouths could be explained by the different sub-bottom pressures of the hydrothermal fluids. The presence of gas bubbling in some chimneys can be attributed to fluids with high pressure, whose decompression near the chimney mouths generates the bubble gas formation. Emissions without bubbling originate from lower sub-bottom pressure fluids, that are not able to generate the gas bubbles, while the apparent inactivity of some chimneys is probably caused by a temporary complete sub-bottom localized depressurization, due to previous partial or total overpressure fluid spilling. However, the highest dissolved benthic fluxes recorded by the benthic chamber and the presence of a high number of chimneys in the Smoking Land suggest that, currently, in this area, the sea water sub-bottom circulation and spilling is the maximum in the Panarea Volcanic Complex.

The formation of the observed chimneys is a result of the Fe^2+^ precipitation of volcanic origin caused by the sea-water with the likely support of microbial activity, as demonstrated by the prevalence of Fe-oxyhydroxide of very recent formation and by the presence of some bacterial-like structures revealed by SEM-EDS images of the collected samples.

The low-temperature Fe-oxyhydroxide precipitates of hydrothermal origin are widely distributed around the Panarea site as well as sulfide ore deposition identified over the eastern slope of the NE elongation of the Panarea platform, to the North of Basiluzzo [16,17,18,29], where they formed different structures and habitats. The North-eastern platform of Basiluzzo islet is characterized, at the depth strata from 70 to 100 m, by compact Fe-oxyhydroxide hardgrounds forming a stopper to the gas and fluid fluxes at the bottom. The presence of hard-impermeable layers of hydrothermal origin off Panarea have been reported by Becke et al. [39] and Price et al. [40] who modeled the deposition processes and their relationships with the microbial communities. As reported by Giacobbe et al. [20] and Esposito et al. [21], the benthic community associated with the Fe-rich crust at these depths is strongly dominated by the tube-dweller amphipod *Ampelisca ledoyeri*.

In the steep slope (starting from 180 m) the fresh semi-consolidated Fe-rich hydrothermal precipitates form numerous small, yellow- to dark red-colored chimneys, better defined as “ferruginous diffusive seeps” [23], due to the apparent lack of fluid emission [17,18,29]. These structures resulted as having been colonized by a few and sparse sessile species [22,23].

Although the described habitats showed a geochemical composition similar to that of the explored vents, they showed differences in hydrothermal dynamics that affect the associated communities.

The living assemblage associated with the hydrothermal vents of the Smoking Land contains no vent-exclusive species and is composed of benthic organisms that Ballesteros et al. [41] classified as part of the communities found in the coralligenous concretion of shallow water on vertical walls in the Mediterranean Sea. The acidic chemical conditions around the chimneys allowed the growth of the algae *Peyssonnelia* sp. that is characterized by a carbonate content lower than the average carbonate content in the encrusting Corallinales [42,43] observed in the no-vent area, and as a result is more tolerant to low aragonite saturation level caused by low pH and high pCO_2_ levels [44]. However, macroalgae were represented by few species among which the most abundant *F*. *petiolata* (Chlorophyta) widely colonized the summit of the vents, decreasing along the chimney walls and disappearing near the sea bottom, where pH reaches the minimum measured values of 6.19–6.96. This distribution pattern is consistent with that observed by Porzio et al [45] along a horizontal pH gradient, in macroalgal communities associated with the shallow water CO_2_ seeps of Ischia Island (southern Tyrrhenian sea), with a loss of macroalgal diversity in the acidified site near the vent and *F*. *petiolata* dominant at a mean pH of 7.8 and absent at a mean pH of 6.7.

The framework created by *Peyssonnelia* sp. on the chimney walls provides a secondary substratum for the settlement of encrusting and sessile organisms, such as hydrozoans and sponges but also bryozoans and tubicolous polychaetes, and numerous microcavities for cryptic species, such as fish of the Gobiidae family. However, scleractinians and large gorgonians, usually abundant in coralligenous communities, were rarely observed. Mollusks were represented by only two species, and echinoderms, largely observed in the surrounding no-vent areas, were occasionally found. These findings agree with Goffredo et al [46] that investigating the effect of pCO_2_ on the abundances of calcified organisms in a shallow water hydrothermal area of Panarea (Bottaro islet), reported that as pCO_2_ increased, the abundances of some species of coral and mollusk severely decreased. Nevertheless, the presence of *Caryophyllia* sp. on some of the explored chimneys with emissions could be explained by the resilience of this and other calcified scleractinians to water acidification, as observed by Rodolfo-Metalpa et al. [47] in an experiment conducted on a rocky seabed of CO_2_ seeps off Ischia. The benthic community associated with the chimneys resulted as being composed of more *taxa* than those observed in the no-vent areas surrounding the Smoking Land. Similar patterns were observed by Morri et al. [48] and Bianchi et al. [49] in epibenthic assemblages associated with the shallow vent area of Paleochori Bay (Milos, Greece), with biodiversity proportionally higher at the sites closest to hydrothermal vents. As suggested by Bianchi et al. [49], and revealed also by Johnson et al. [50] in the hydrothermal vents of Vulcano, another island of the Aeolian archipelago, it is possible to hypothesize that the high pCO_2_ levels measured in seawater stimulates the growth of some benthic microalgae and enhances the rate of photosynthesis in primary producers promoting the primary productivity that, coupled with the bacterial chemosynthetic production typically observed in hydrothermal habitats, increases the amount and variety of organic matter available to filter and suspension-feeders and other consumers such as grazers and fish. These were found aggregating close to strong gas plumes and wide bacterial mats, enhancing the overall species richness in communities associated with the vents. These findings agree with Cardigos et al. [51] that observed fish using gas plumes as feeding stations as the water drawn in by the rising gas bringing food.

Moreover, as observed in the Smoking Land, sponges are considered one of the most important components of epibenthic communities associated with shallow hydrothermal vents. Species of this phylum were abundantly found in the hydrothermally influenced submarine cave of Palinuro (South of Italy) [52,53] and at Milos [54], in the Mediterranean Sea, but also in SW Pacific, in hydrothermally influenced water off Ambitle Island, Papua New Guinea [55] and Indonesia [56].

All the characteristics described in the present study, the metal-rich geochemical composition of the hydrothermal structures, the supposed presence of chemosynthetic bacteria and the emissions of fluids enriched in carbon dioxide and toxic chemicals, make the Smoking Land, commercially and scientifically interesting. Indeed, this chimney field, as well as other vent systems, can be considered as a potential mineral and genetic source for societal needs, but also as a natural laboratory to test hypotheses about the effect of global change on marine biodiversity and ecosystem functioning [57–59].

Destructive commercial (tourism, non-harvest genetic resource development, mineral exploration, fishing) and scientific (deposits and structures sampling) activities can have detrimental impacts on morphological, geochemical and biological dynamics of vent ecosystems, therefore, considering the importance and the peculiarity of these habitats, some management indications could be useful to guarantee their protection.

Mitigation measures have to limit and minimize the impacts, allowing not only the protection but also the restoration or rehabilitation of the vent ecosystems. Underlying mitigation frameworks must include multi-pronged information on the natural ecosystem, including its biogeographic context, geochemistry, mineralogy, biodiversity, community and trophic structure, connectivity, ecosystem services, disturbance regimes and community dynamics, but also the most impacted sites and the most sensitive organisms [58,60].

Many countries have created Marine Protected Areas (MPAs) to protect deep-sea vent ecosystems [61], including Canada (Endeavour Hydrothermal Vents MPA), Mexico (Guaymas Basin and Eastern Pacific Rise Hydrothermal Vents Sanctuary), Portugal (Azores Hydrothermal Vent MPAs), and the United States (Mariana Trench National Monument). MPAs that include hydrothermal vents can implement avoidance measures by monitoring and managing human activities. A MPA designation provides the basis to manage the area comprehensively, to conserve and protect the ecological integrity through the identification of integral reserve areas that include the most important geochemical and morphological structures of the vents system and where only observational activities are permitted.

Moreover, to minimize impacts of mineral extraction and their application to a future extractive operation, the voluntary Code for Environmental Management of Marine Mining consider the establishment of un-mined biological corridors (temporary refuges) within a mine site to aid in recovery of the biota and site rehabilitation. In addition, three-dimensional structures (artificial substrates) can be deployed to provide topographic relief and structural stability for developing sulfide deposits following mining and relocation of animals within the site to facilitate re-establishment of characteristic invertebrates [62].

To date, the Smoking Land described here seems to not be particularly affected by human impacts, although the presence of some lost fishing gears on the bottom was also observed during ROV explorations. Given that the Aeolian Islands are characterized by intense seasonal tourism and the presence of an important artisanal fishery [63], the area should be preserved by potential impacts generated from these activities on the fragile hydrothermal structures and the associated fauna. To this end, the future establishment of a Marine Protected Area in the Aeolian archipelago, according to Italian Law 979/82, art. 31, could take into account the preservation of the Smoking Land during the zonation planning process.

Moreover, the Panarea Volcanic Complex is visited by one to several marine research expeditions every year. Biological and geological samples are collected and instrumentation is left behind. The pressure exerted by these activities may grow over the long-term with technological advances and increased scientific and public interest, causing the habitat degradation from physical damage. Concern about these impacts prompted development of a voluntary code of conduct for scientific research at vents that emphasizes avoidance of activities that might have long-lasting and deleterious effects [64].

Voluntary actions would require greater coordination and collaboration between marine scientific institutes in sharing information, awareness and good will, to alleviate collecting pressure at the most popular sites and to devote dive time to exploring new sites for collection [65]. The principles upon which a voluntary code is based must be known to all the participants and could include: the duty to inform other researchers about the periods in which investigations will take place, the investigated sites and the type of activities that will be carried out during scientific surveys; the coordination with other institutes to avoid the overlapping of scientific explorations and samplings in the same sites; the maximization of the sampling efficiency through the reduction of discards and the development of non-destructive techniques. Such a code of conduct would help to guide researchers and to provide a reference point against which they can judge their own conduct and the conduct of their peers [60].

In the study area, where a plan to manage research involving hydrothermal systems does not exists, a code of conduct could be particularly useful to minimize the conflicts and environmental impacts that scientific research activities can pose to hydrothermal vents and their associated biological communities, and it will be also applied as an intermediate step towards the application of more detailed rules.

Our results, other than providing a first description of the geological and geochemical features of the Smoking Land and the associated fauna, evidence the complex scenario of the influencing factors that vents exert on the biota in shallow-water hydrothermal systems and give some indication to manage human activities affecting these particular ecosystems. However, further investigations will allow the definition of the geochemical features of the fluids circulating over the area and to understand the hydrothermal dynamics, the productivity and the trophic structure associated with the studied vents and consequently inform adaptive management.

## Supporting information

S1 Dataset Trace elements and DIC concentrations measured in water samples collected inside the benthic chamber and physico-chemical values measured by the multiparameter probe inside the benthic chamber in each deployment. (XLSX)

S2 Dataset PRIMER matrix with species presence/absence data according to the three considered categories of area. (XLSX)
